# Influence of Lidocaine including Buprenorphine for Postoperative Analgesia after the Extraction of Mandibular Third Molars: A Randomized Controlled, Double-Blind, Split-Mouth Study

**DOI:** 10.1155/2021/7097948

**Published:** 2021-11-13

**Authors:** Nimrat K. Jawanda, Anand Shukla, Anupam Singh, Kalyana C. Pentapati, Srikanth Gadicherla

**Affiliations:** ^1^Department of Oral & Maxillofacial Surgery, Manipal College of Dental Sciences, Manipal Academy of Higher Sciences (MAHE), Manipal, Karnataka, India; ^2^Department of Public Health Dentistry, Manipal College of Dental Sciences, Manipal Academy of Higher Sciences (MAHE), Manipal, Karnataka, India

## Abstract

**Background:**

The presence of opioid receptors around the peripheral nerves offers the possibility of providing postoperative analgesia, thereby encouraging the study of the effect of opioids in combination with local anesthesia (LA). Studies have also reported the efficacy of peripherally administered opioids in achieving adequate analgesia in regions with inflammation. Applying the concept of peripheral opioid receptors, our study aimed to evaluate the effectiveness of opioid analgesia in managing postoperative pain. The split-mouth study was carried out to evaluate the efficacy of buprenorphine added to lidocaine 2% in providing postoperative analgesia after the surgical extraction of the impacted mandibular third molar.

**Materials and Methods:**

We conducted a randomized, double-blinded, split-mouth trial among 21 patients with impacted mandibular third molars bilaterally. In all patients, bilateral impacted mandibular third molars were extracted at different periods. The primary outcomes assessed were postoperative analgesia by the VAS score and the number of rescue analgesics consumed by patients at 24, 48, and 72 hours of interval via a questionnaire.

**Results:**

There was a statistical significant difference in postoperative analgesia duration at 24 (*P* = 0.012) and 48 hours (*P* = 0.024), respectively, between the test and control group. Even though the mean number of rescue analgesics consumed by the test group was less than that of the control group, no significant difference was seen.

**Conclusion:**

Buprenorphine added to lidocaine 2% showed a minimal decrease in the pain score and duration of postoperative analgesia with no difference in the frequency of rescue analgesics consumed between the test and control.

## 1. Introduction

The impacted mandibular third molars in the oral cavity requiring surgical removal predispose the patient to pain and discomfort. While surgical extraction of the third molars of the lower jaw is a common procedure, pain management is the biggest apprehension for the patients. While intraoperative pain is well managed under local anesthesia (LA), the major problem lies postoperatively when the effect of the anesthesia dissipates [1]. The pain and discomfort postoperatively can make the patient's entire experience of the treatment unpleasant, thus making the management of pain a critical aspect [2, 3].

Extraction of teeth is generally done under the effect of a local anesthetic agent, commonly being lidocaine 2% with epinephrine 1 : 2,00,000. The duration of the effect of the local agent is approximately 40–60 minutes which is the duration of the surgical procedure. As soon as the effect of the local anesthetic agent has dissipated, the patient begins to develop pain. To overcome this, the patient consumes analgesics postoperatively [4]. The commonly used analgesics are nonsteroidal anti-inflammatory drugs (NSAIDs) like ibuprofen, aspirin, and diclofenac or centrally acting opioids like morphine. These drugs effectively manage pain postoperatively; however, they are individually associated with various adverse effects. NSAIDs are associated with systemic effects such as peptic ulcers, platelet dysfunction, and renal and liver dysfunction. On the contrary, opioids that are *µ*-agonists with no direct organ damage offer an effective alternative for pain management. However, opioids may also cause central effects such as dizziness, fatigue, respiratory depression, hypotension, mental clouding, and vomiting [5].

This led to the understanding of a drug, buprenorphine hydrochloride, with a potent analgesic effect and close to no adverse systemic effects. Buprenorphine hydrochloride is a synthetic opioid having *µ*-agonistic, *κ*-antagonistic, and antihyperalgesia effects. The pharmacological effects of buprenorphine are generally alike to morphine (*μ*-opioid receptor agonist) and are said to be 20–25 times more potent than morphine (buprenorphine 0.3mg is as equipotent as morphine 10mg) with a rapid onset and longer duration of action [6].

In contrast to the other opioids, buprenorphine appears to have far fewer adverse effects such as tolerance and hyperalgesia. The use of systemically suboptimal dose of buprenorphine in the area of peripheral nerve endings resulting in analgesic effect has helped prove the theory of peripheral action of opioids [7–10]. It has also been established that the local administration of opioids is more effective in providing longer pain relief, due to their ability to dissociate at a slower rate, and without any central side effects associated with opioids [11, 12]. Another unique property of opioid *μ*-receptors is their upregulation with change in pH of the surrounding environment, such as the areas of inflammation, making these opioid agents more effective in the intraoral surgical sites which are generally inflamed and infected [13, 14].

Previous trials on the use of buprenorphine with LA have shown a marked reduction in postoperative analgesia [15–18]. These studies were parallel arm trials that used different individuals for the test group and control group. Pain, being a subjective indicator, may vary widely between patients. Hence, we aimed to evaluate the efficacy of lidocaine with or without buprenorphine for postsurgical analgesia after the removal of mandibular third molars in the same patients by a split-mouth study model.

## 2. Materials and Methods

The study followed the Declaration of Helsinki on the medical protocol and ethics, the institutional ethics committee of Kasturba Hospital, Manipal (KH-IEC: 97/2019), approved the protocol, and prior informed consent was obtained from all patients. The trial was registered with the Clinical Trial Registry of India (CTRI/2019/08/020631). A prospective, randomized, double-blind, split-mouth trial was conducted in 21 patients who required bilateral impacted third molars' removal. The study was conducted from January 2019 to December 2020 at the Department of Oral and Maxillofacial Surgery.

Patients who required bilateral impacted mandibular third molar extractions by the transalveolar method and aged between 18 and 40 years without any comorbidities with Pederson's difficulty index ≤6 were included [19]. Patients with a history of local anesthetic drug allergy or a history of substance abuse or the patients who had consumed analgesics 6 hours before the procedure were excluded from the study.

The study had a split-mouth design; each patient served as their own control. However, randomization was used using the coin toss method to determine which side received test or control intervention. The patient was unaware of the intervention being done, and the operator who administered LA was also blinded.

### 2.1. Method of Preparation of the Solution

Two solutions were prepared as a double-blinded study; solution A contained a modified solution of lidocaine 2% with epinephrine 1 : 20,000 mixed with buprenorphine, and solution B contained lidocaine 2% with epinephrine 1 : 2,00,000. The concentration of buprenorphine added to lidocaine 2% was 1 ml of 0.3 mg in a 30 ml vial (0.01 mg/ml). The dental nurse prepared the solution after she was handed over the coin by the respective patient. The maximum amount of LA administered was 3 ml. The amount of buprenorphine in 3 ml of LA administered was 0.03 mg (0.01 mg/ml × 3 ml).

### 2.2. Administration of Local Anesthesia

The standard technique for the inferior alveolar nerve (IAN) block was used to administer the LA by a single operator, NJ. All patients received a maximum of 3 ml of the solution (2 ml inferior alveolar nerve block + 0.5 ml lingual nerve block + 0.5 ml long buccal nerve block). The side of the mouth which received solution A was categorized as the test group, and the side of the mouth which received solution B was categorized as the control group.

### 2.3. Study Paradigm

The position of the tooth to be extracted was classified as per Pederson's difficulty index of the impacted third molar, which was assessed on a preoperative OPG of the patient [19]. The difficulty index of the tooth was assessed by two observers SG and AS independently. The patients were included in the study only if the difficulty index of the impacted tooth was assessed to ≤6 by both observers. As per the standard surgical procedure, a complete systemic evaluation was done before starting the procedure.

The extraction was performed under LA under aseptic precautions as per the standard protocol by a single operator NJ. The onset and effect of the anesthesia were assessed as per the objective signs and symptoms for the inferior alveolar nerve block. Sutures were placed after completion of the extraction and achieving hemostasis. After extraction, the patient was prescribed antibiotic amoxicillin and potassium clavulanate (500 + 125 mg) combination three times per day for three days and an anti-inflammatory-analgesic combination of diclofenac 50 mg with paracetamol 325 mg as per the requirement, subject to not exceeding three tablets in a day.

### 2.4. Pain Assessment

The primary outcomes assessed were postoperative analgesia and the frequency and number of rescue analgesics consumed by the patient. Pain was quantified using the Visual Analogue Scale (VAS) and documented by the patient before taking the rescue analgesic. A 10 cm VAS assessed the postoperative pain, ranging from 0 to 10, with 0 indicating no pain and 10 indicating the most severe pain. In addition, the patient documented the frequency and number of rescue analgesics consumed, which was collected on their follow-up visit. For this, the patients were given the preprinted forms and were instructed to mark their VAS score for pain at 24, 48, and 72 hours of intervals. Similarly, the number of analgesics consumed along with time was also mentioned on the provided form. A follow-up reminder was sent to each patient telephonically.

### 2.5. Data Analysis

The analysis was done using SPSS version 20 (IBM Corp. Released 2011. IBM SPSS Statistics for Windows, Version 20.0. Armonk, NY: IBM Corp.). A *p* value <0.05 was considered statistically significant. Comparison of pain scores and the number of rescue analgesics between the test and control was done using Wilcoxon's signed-rank test.

## 3. Results

**Figure 1:**
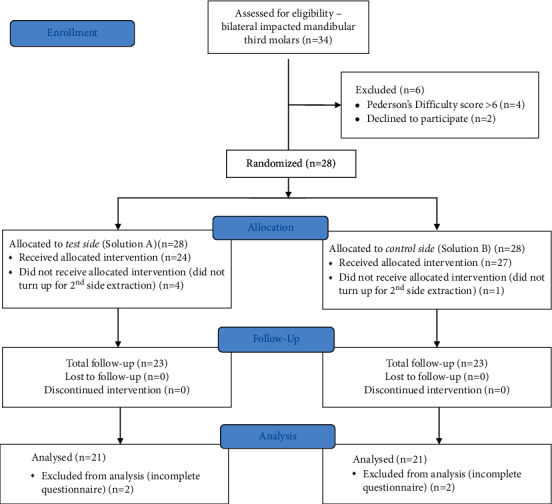
CONSORT flowchart showing the progress of participants included in the randomized controlled trial.

The initial 28 patients had five patients excluded because they underwent only single-side extraction. Additionally, two patients were excluded because of an incomplete questionnaire. The final study comprised 21 patients, of which 11 were males and 10 were females (Figure 1). The mean age of the patients was 24.33 ± 5.34 (range: 19 to 38 years). No intraoperative or postoperative complication was encountered for any of the patients.

 
*Effect on the Pain Score*. The mean pain score on the test side was significantly lower than on the control side at 24 and 48 hours (*P* = 0.012 and 0.024). Similarly, lower pain scores were noted on the test side than the control side at 72 hours; however, at 72 hours, the difference was not statistically significant (*P* = 0.064) (Table 1).
 
*Frequency of Rescue Analgesics Consumed*. The mean number of rescue analgesics was lower on the test side than on the control side. However, on comparing the test with the control, there were no significant differences between 24, 48, and 72 hours (*P* = 0.132, 0.096 and 0.058) (Table 2).

## 4. Discussion

The surgical procedure involves a mucosal incision to raise a mucoperiosteal flap, exposing the underlying impacted tooth, thus making it an invasive procedure. Tissue damage initiates the release of inflammatory mediators such as bradykinin and histamine. These inflammatory mediators act on nociceptors. Consequently, pain, inflammation in the submandibular area, and the submasseteric region with trismus are likely the outcomes [3].

Postoperatively, analgesics and anti-inflammatory drugs are prescribed, the most common being NSAIDs (nonsteroidal anti-inflammatory drugs). Despite their analgesic effect, they potentially cause side effects, including GI disturbances and renal toxicity, making them undesirable. Opioid analgesics, on the contrary, are a primary line of treatment for managing debilitating conditions. They are not known to cause direct harm to organs in low doses [20]. However, on systemic administration, they are known to cause central effects such as dizziness, fatigue, respiratory depression, hypotension, mental clouding, vomiting, and increased pressure in the biliary tract [21].

The presence of opioid receptors around the peripheral nerves exposed the possibility of achieving postoperative analgesia, thereby encouraging studies of the effect of opioids in combination with LA [22]. However, due to various central effects reported by the use of morphine, studies were conducted to compare the efficacy of a more potent opioid that is a partial agonist of the *δ*-opioid receptor and antagonist of the *κ*-opioid receptor and shows lesser systemic side effects. Buprenorphine, a weak *μ*-receptor agonist and *κ*-receptor antagonist, was compared with morphine and produced a longer duration of analgesia with lesser side effects [23–25].

Recent advances allowed for the supplementation of LA with adjuvants such as steroids, which prolonged its postoperative analgesic effect without unduly extending the anesthetic effect and were efficient in inflammatory conditions [26, 27]. Similarly, a study for assessing the effect of the combination of buprenorphine with 0.5% bupivacaine, a long-acting local anesthetic agent, was conducted by Modi et al., which showed a significant benefit in postsurgical pain management. However, the major drawback of this study was that since bupivacaine itself is a long-acting anesthetic agent, it might mask the opioid-induced analgesia, resulting in a biased outcome. The possibility of cardiotoxicity of bupivacaine makes it an undesirable routine anesthetic agent [15].

Numerous studies have shown a beneficial effect of adding buprenorphine to various local anesthetic agents while administering regional blocks [7, 11, 28]. A meta-analysis by Schnabel et al. evaluating the efficacy and safety of buprenorphine in regional blocks showed significant postoperative analgesia. However, it was also found to be associated with postoperative nausea and vomiting [10]. Based on our literature search, only two studies have been published regarding the use of buprenorphine with LA for intraoral block-related surgical procedures [16, 17]. However, other studies comparing the efficacy of tramadol hydrochloride as an anesthetic agent compared to lidocaine hydrochloride for the maxillary infiltration technique and orthodontic tooth extraction procedure have shown tramadol hydrochloride to be an effective alternative local anesthetic and with better postoperative analgesic property [29, 30] 

Our study showed a significant pain reduction score over a 48-hour period in the test group, in which buprenorphine was administered along with lidocaine 2% for the IAN block compared to the control group in which unmodified local anesthetic solution was administered. This finding was consistent with the findings in the other two studies. Chhabra et al., in their study, reported that patients remained pain-free for 12 hours on administering buprenorphine-mixed LA solution [16], while Kumar et al., in their study, reported a pain-free period of up to 36 hours after the procedure [17]. This prolonged duration of postoperative analgesia, denoted by the low VAS pain scores, can be attributed to the highly lipophilic nature and slow dissociation of buprenorphine from the opioid receptors. The addition of low-dose buprenorphine (0.03 mg) to LA and its significant effect on the duration of analgesia and severity of pain verify the concept of the existence of peripheral opioid receptors [26, 27].

Another parameter assessed in our study was the number of rescue analgesics consumed by the patient postoperatively. Even though the mean number of rescue analgesics was lesser in the test group, it was not statistically significant. In contrast, in both studies conducted by Chhabra et al. and Kumar et al., a significantly lesser number of rescue analgesics were consumed by the patients in the test group [16, 17]. This variation in the result can be attributed to different pain thresholds of different patients. However, the mean number of rescue analgesics consumed in our study over 48 hours (1.79) was found to be similar to the ones reported by Chhabra et al. (1.05) and Kumar et al. (1.86).

The systematic review had considerable heterogeneity in both studies conducted regarding the study design and parameters assessed [18]. Chhabra et al., in their study, included the patients with impactions of varying difficulties [16]. The surgical technique for difficult impaction can be more traumatic, thereby potentially affecting the degree of postoperative pain. Additionally, they did not specify the number of operators carrying out the extraction procedure, which could also possibly confound the results owing to the difference in surgical techniques. In our study, we tried to standardize the intervention as a single operator only carried out the surgical procedure. The study was limited only to easy or moderate impaction, thereby reducing the confounding factors affecting the study outcomes.

Kumar et al., in their study, included all the patients undergoing any form of intraoral surgical procedure which required nerve block administration. Furthermore, there was no mention regarding blinding of patients or operators in their study [17]. Both these factors could introduce bias, and they affected the result of the study. This drawback was overcome in our study as we limited the intervention only to the surgical extraction of the impacted mandibular third molar. Also, by concealed allocation of the patient by the coin toss system to the test group or control group, we tried to minimize the bias in our study.

Pain is a subjective experience, and perception of its intensity can vary with different patients. We designed a split-mouth study that included patients with the bilateral easy to moderate grade of mandibular third molar impaction to overcome this. The same patient was included in both test and control groups and assessed for their pain by the VAS chart. We tried to minimize or negate this subjective evaluation of pain perception and psychological differences between the tested individuals, affecting the results.

No adverse effects were reported; this could be attributed to the low concentration of buprenorphine, i.e., 0.03 mg used peripherally. These results were similar to the other documented studies on the use of buprenorphine with LA for intraoral use. However, these reports regarding systemic side effects are in contrast to the one published in the systematic review regarding the use of buprenorphine for regional blocks, which had been attributed to a comparatively higher dose of buprenorphine which was administered [10]. Thus, we can infer that 0.03 mg of buprenorphine is effective and safe when administered for intraoral block techniques.

In our study, addition of buprenorphine to lidocaine reduced the pain severity postoperatively and extended the postoperative analgesia duration; the study was constrained by not including patients allergic to LA and limited to patients with a narrow range of the difficulty index between bilateral impacted third molars.

## 5. Conclusion

Administration of local anesthetic solution mixed with buprenorphine for intraoral block-related procedures effectively reduces postoperative pain, with no significant side effects. The current study demonstrated a significant reduction in the mean pain score in the first 48 hours, in patients who were administered lidocaine with buprenorphine. However, there was no significant difference in the number of rescue analgesics consumed between the test and the control interventions. Furthermore, addition of buprenorphine for administration at the local site did not show any significant adverse effect. Hence, we can conclude that administration of local anesthetic solution mixed with buprenorphine has beneficial effects in prolonging postoperative analgesia after mandibular third molar extraction. Similar studies can be carried out to assess its efficacy in various other intraoral minor surgical procedures.

## Figures and Tables

**Table 1:** Comparison of the mean pain scores between test and control interventions.

Pain score	Control		Test		*P* value
	Mean	SD	Mean	SD	
24 hours	5.00	3.11	3.71	2.95	0.012; Sig.
48 hours	4.21	3.02	3.07	2.46	0.024; Sig.
72 hours	3.43	2.53	2.50	2.50	0.064; NS

Sig.: significant; NS: nonsignificant.

**Table 2:** Comparison of the mean number of rescue analgesics between test and control interventions.

Number of rescue analgesics	Control		Test		*P* value
	Mean	SD	Mean	SD	
24 hours	2.14	0.66	1.79	0.89	0.132; NS
48 hours	2.14	0.86	1.79	0.80	0.096; NS
72 hours	1.93	0.92	1.50	0.76	0.058; NS

NS: nonsignificant.

## Data Availability

The data used to support the findings of this study are available from the corresponding author upon request.
